# Correlation between evolution of inclusions and pitting corrosion in 304 stainless steel with yttrium addition

**DOI:** 10.1038/s41598-018-23273-x

**Published:** 2018-03-19

**Authors:** Weining Shi, Shufeng Yang, Jingshe Li

**Affiliations:** School of Metallurgical and Ecological Engineering, University of Science and Technology Beijing, Beijing, 100083 China

## Abstract

Effects of the evolution of inclusions on the pitting corrosion resistance of 304 stainless steel with different contents of the rare-earth element yttrium (Y) were studied using thermodynamic calculations, accelerated immersion tests, and electrochemical measurements. The experimental results showed that regular Y_2_O_3_ inclusions demonstrated the best pitting resistance, followed in sequence by (Al,Mn)O inclusions, the composite inclusions, and irregular Y_2_O_3_ inclusions. The pitting resistance first decreased, then increased, and then decreased again with increasing Y content, because sulfide inclusions were easily generated when the Y content was low and YN inclusions were easily generated at higher Y contents. The best pitting corrosion resistance was obtained for 304 stainless steel with addition of 0.019% Y.

## Introduction

As very important strategic resources, rare-earth elements are widely used in the petroleum, chemical, metallurgical, textile, ceramics, and glass industries, and as permanent magnetic materials. Addition of rare-earth elements at an appropriate content is beneficial to enhancing the mechanical properties of metallic materials by refining the grains, improving wear and corrosion resistance, and improving plasticity, strength, grain boundary strength, and dislocation movement^1,2^. Zhao *et al*. found that appropriate Y content promoted the formation of fine carbides, which prevented the migration of grain boundaries, impeded grain growth in the recrystallization process, and refined the grains^3^. More importantly, Y also benefitted resistance to overall corrosion or pitting^4–7^.

Rare earths enhance overall corrosion resistance by changing the inner structure and the chemical composition and structure of the material surface, and promote and stabilize the formation of uniform and compact surface films^8–11^. Liu *et al*. suggested that increasing Y content ensured a reticular structure in the Y-enriched area, which enhanced corrosion resistance, while other structures in the Y-enriched area induced galvanic corrosion^12^. Riffard *et al*. showed that implanting Y into the matrix surface or use of an Y sol–gel coating could improve the oxidation resistance of AISI 304 stainless steel^13,14^. Li *et al*. studied the corrosion behaviour of AZ61 Mg alloy by adding 0–0.9 mass% Y and showed that Y treatment not only refined grains and precipitates, but promoted the formation of a passivation film^15^. Wang *et al*. found that the surface film of 09CrCuSb alloy with Y treatment was uniform and compact, which improved the properties of the corrosion product^16^. Wang *et al*. demonstrated that the passivation film on 304 stainless steel containing Y was stabilized, improving its resistance to mechanical failure^17^.

Pitting corrosion resistance is enhanced because rare earths change the morphology, size, type, composition, and distribution of inclusions in the matrix. Cai *et al*. found that adding Ce to steel enabled easy transformation of MnS inclusions into multiphase inclusions containing Ce_2_O_2_S, which improved the corrosion resistance of 202 stainless steel; however, inappropriate Ce addition produced a brittle secondary phase that deteriorated pitting corrosion resistance^18^. Kim *et al*. illustrated that the addition of rare-earth metals to a base alloy led to the formation of (Mn,Cr,Si,Al,Ce) and (Mn,Cr,Si,Ce) oxides, which improved resistance to pitting corrosion^4^.

Stainless steel produced by smelting has good corrosion resistance, so many scholars have endeavoured to improve this property. The evolution of inclusions and microstructures in stainless steel with rare-earth additions has been paid much attention^19–22^. Chen *et al*. found that many fine Y-enriched oxide inclusions were non-uniformly distributed in 21Cr–11Ni austenitic stainless steel containing Y and that segregation of sulfur into grain boundaries was reduced^23^. Kim *et al*. illustrated that adding a rare-earth metal into duplex stainless steel rendered the inclusions smaller and their shape became spherical, which improved the pitting corrosion resistance^4^. With the progress of smelting technology, sulfide inclusions have been largely removed and gradually replaced by oxide inclusions, which greatly enhances pitting corrosion resistance. Jun *et al*. argued that conditions where the number of sulfide inclusions was smaller than that of the oxide inclusions and the size and distribution of oxide inclusions were small and dispersed would improve the pitting corrosion resistance of high clean 304 stainless steel^24^. There are, however, few studies focusing on modifying oxide inclusions by adding rare-earth Y to improve the pitting corrosion resistance of clean 304 stainless steel.

In this study, we determined the effects of the evolution of inclusions on the pitting corrosion resistance of 304 stainless steel with Y addition using thermodynamic calculations, potentiodynamic polarization and immersion tests, and scanning electron microscopy with energy-dispersive spectroscopy (SEM–EDS) analysis of inclusions.

## Methods

### Materials and specimen preparation

**Figure 1:**
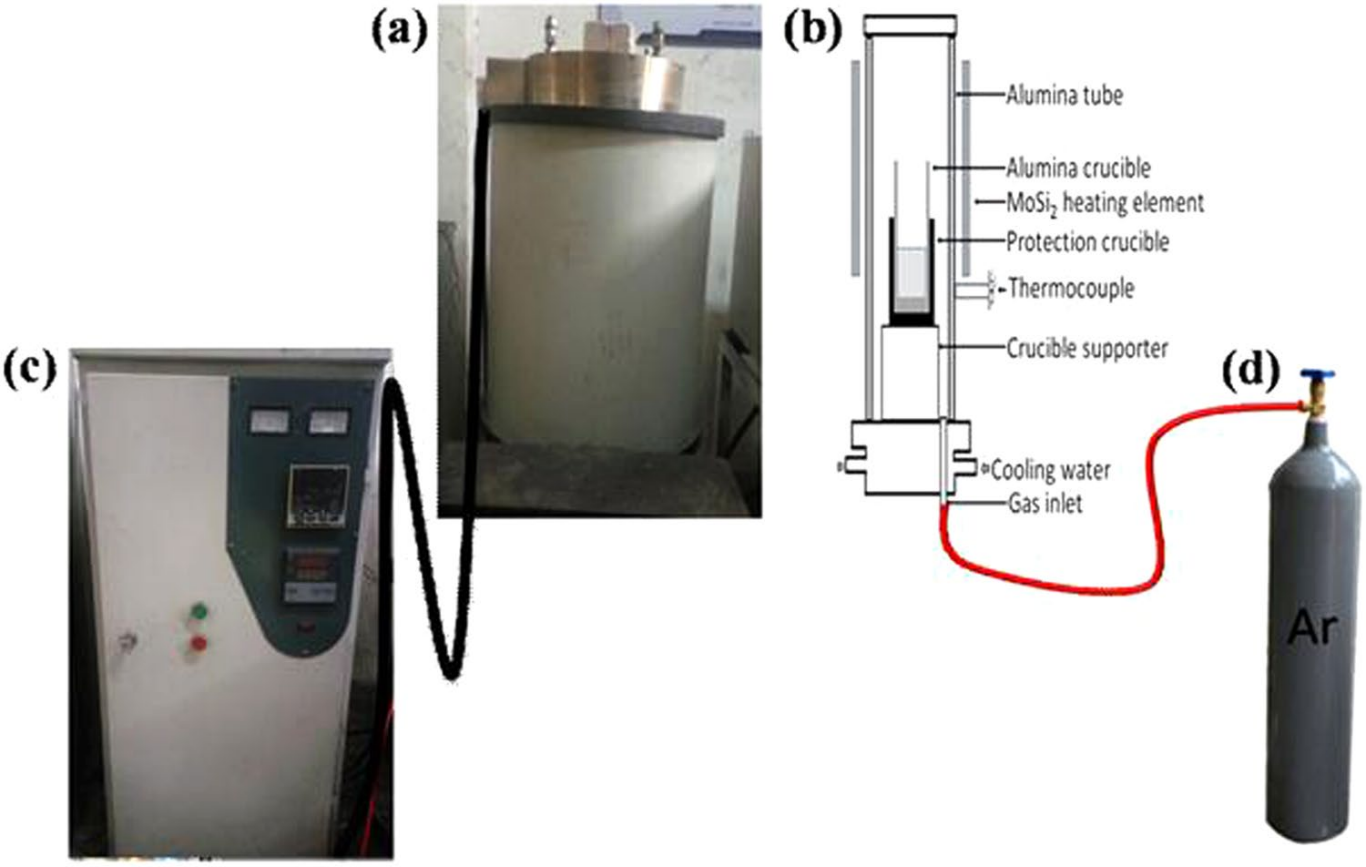
The Si-Mo heating electric resistance furnace.

The raw material used in this study was 304 stainless steel of the chemical composition given in Table 1. The experimental alloys were prepared using a Si–Mo electrical resistance-heated furnace. The Y contents of the experimental alloys were 0, 0.007%, 0.013%, 0.019%, and 0.049%. Figure 1 shows the Si–Mo furnace with (a): resistance furnace body; (b): body sketch; (c): program console; (d): argon tank. A smelting process was executed by switching on the flows of argon gas and cooling water, covering the furnace mouth with refractory bricks, and then programming an appropriate procedure into the console. After cooling of the furnace, the specimen was not heat treated in any other way. Specimens of the 304 stainless steel with different Y contents were used for counting inclusions and for the immersion and electrochemical tests. To avoid surface defects, the test surfaces were ground with 2000 grit silicon carbide paper and polished with 0.5 μm diamond paste, then rinsed with deionized water, degreased in alcohol, and dried immediately.

**Table 1:** Chemical composition of 304 stainless steel specimens (mass%).

C	Si	Mn	P	S	Cr	Ni	Mo	Cu	Al	O	N	Ca	Fe
0.0511	0.4016	1.2005	0.0334	0.0019	18.0614	8.048	0.0198	0.0391	0.0132	0.0031	0.0377	0.002	Balance

### Determination of inclusion evolution process

The types and sizes of inclusions in the 304 stainless steel specimens with different Y contents were observed and analysed by SEM–EDS. Several hundred inclusions were randomly selected and classified according to their distribution on a test surface using *Factsage 7.0* software to reveal their evolution. After choosing the database, the *Equilib* software module was selected, the alloy compositions were input (where the Y content was set as a variable), the desired phase (as determined by SEM) was selected, and the compositions of inclusions with increasing Y contents in the matrix were calculated.

### Electrochemical measurements

To reveal the pitting trends of 304 stainless steel with different Y contents, potentiodynamic polarization tests were conducted using a three-electrode configuration in 3.5% NaCl solution at 298 K. A copper wire was attached to the rear side of each specimen and mounted in an epoxy resin and the test surface was ground and polished. A platinum sheet and a saturated calomel electrode (SCE) were used as the counter and reference electrodes, respectively. The working electrode was the specimen, the exposed area (test surface) of which was 1 cm2. Potentiodynamic polarization tests were carried out using a Solartron 1287 power supply. Tests were conducted in the potential range of −0.5 V_SCE_ to + 0 V_SCE_ at a scanning rate of 3.8 × 10^−4^ V/s.

### Immersion tests

To clarify the correlation between inclusions in 304 stainless steel with different Y contents and pitting corrosion, immersion tests were carried out for times of 0 s, 5 s, 10 s, 20 s, 30 s, 1 min, 5 min, 10 min, 15 min, and 18 min at 298 K, following which the corrosion morphology of the inclusions was observed *in situ*. The test solution comprised 350 ml deionized water with 69.9 g FeCl_3_∙6H_2_O and 20 ml HCl (36–38 mass%). Each specimen was sealed with epoxy resin except for the test surface to avoid formation of porosity and cracks around the surface. After reaching the set immersion time, the specimen was immediately removed from the test solution, rinsed, and dried. SEM–EDS was used for observing the corrosion morphology and analysing the compositions of inclusions after different immersion times.

## Results

### Counting inclusions and thermodynamic calculations

Five compositions of 304 stainless steel with different Y contents were obtained by tube furnace smelting. The principle of counting related to the type and size of inclusions after cooling in the furnace. The results of the thermodynamic calculations explained the evolution of inclusions with increasing Y content.

**Figure 2:**
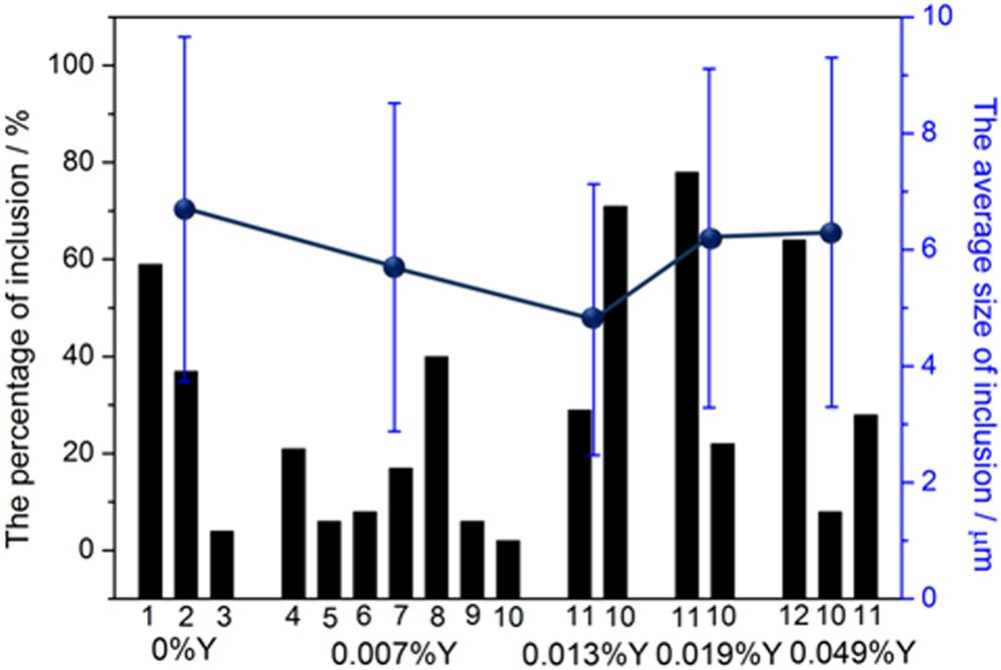
Distribution of inclusions in 304 stainless steel with different Y contents. 1: (Al,Mn)O inclusions, 2: (Al,Mn,Si)O inclusions wrapped in (Al,Mn)O inclusions, 3: (Al,Mn,Si,Ca)O inclusions, 4: (Al,Y)O inclusions wrapped in (Al,Y)_x_(SO)_y_ inclusions, 5: (Al,Y)_x_(SO)_y_ inclusions wrapped in MnS inclusions, 6: (Al,Y,Si)O inclusions, 7: (Y,Mn)_x_(SO)_y_ inclusions wrapped in MnS inclusions, 8: MnS inclusions, 9: CaO inclusions, 10: Irregular-Y_2_O_3_ inclusions, 11: Regular-Y_2_O_3_ inclusions, 12: YN inclusions.

The statistical results for inclusions in 304 stainless steel with different Y contents are shown in Fig. 2. When the Y content was zero, the proportion of (Al,Mn)O inclusions was largest, followed by those of (Al,Mn,Si)O wrapped in (Al,Mn)O. When the Y content was 0.007%, the inclusions were mainly MnS, followed by (Al,Y)O wrapped in (Al,Y)_*x*_(SO)_*y*_ and (Y,Mn)_*x*_(SO)_*y*_ wrapped in MnS. Only two types of inclusions could be found in the steel matrix when the Y content increased to 0.013%: a larger proportion of irregular Y_2_O_3_ inclusions and a smaller proportion of regular Y_2_O_3_ inclusions. The proportion of irregular Y_2_O_3_ inclusions was significantly smaller than that of regular Y_2_O_3_ inclusions when the Y content reached 0.019%. YN inclusions mainly presented in 304 stainless steel containing 0.049% Y, followed by regular Y_2_O_3_ inclusions and then irregular Y_2_O_3_ inclusions. The average size of the inclusions decreased and then increased with increasing Y content. Inclusions with the smallest average size were found in 304 stainless steel containing 0.013% Y.

**Figure 3:**
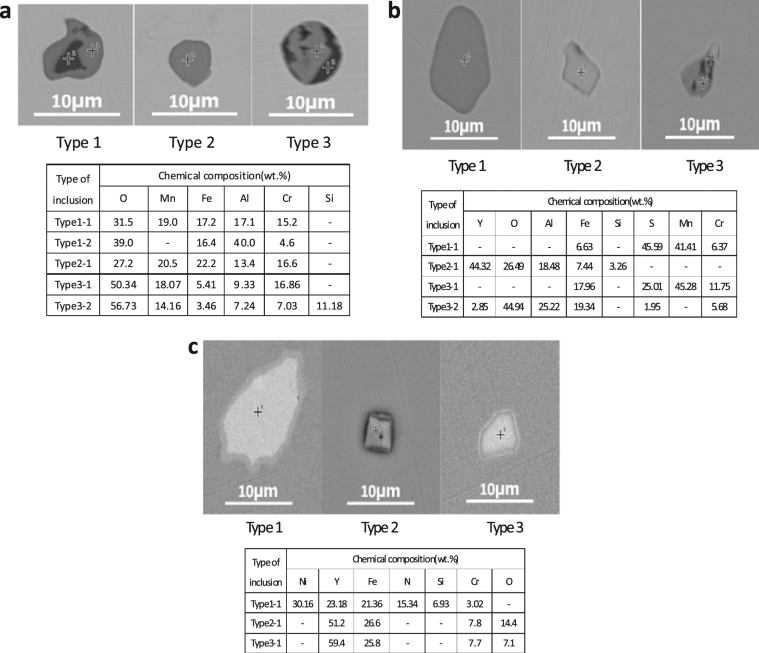
The morphology of main inclusions in 304 stainless steel with different Y contents.

The morphologies of the main inclusions in 304 stainless steel with different Y contents are shown in Fig. 3, where **a**, **b**, and **c** denote the main inclusions in steel matrices containing 0%, 0.007%, and above 0.013% Y, respectively.

**Figure 4:**
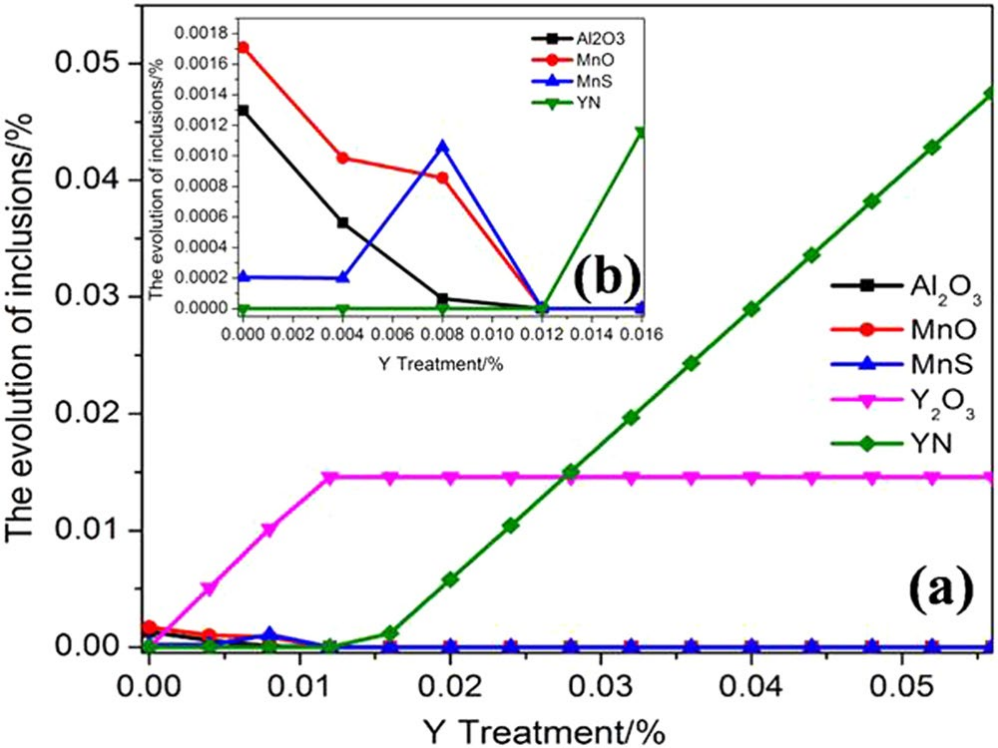
Thermodynamic calculation results. (**a**) Y content range 0–0.05%, (**b**) Y content range 0–0.016%.

The results of the thermodynamic calculations (calculated by *Factsage 7.0*) are shown in Fig. 4. Figure 4a shows that the proportion of Y_2_O_3_ inclusions gradually increased and reached a peak at ~0.012% Y, while YN inclusions gradually increased. Figure 4b shows that the proportions of Al_2_O_3_ and MnO inclusions decreased while those of MnS increased and then decreased. YN inclusions were surprisingly generated at ~0.012% Y. The Al_2_O_3_ and MnO components reacted with Y to generate Y_2_O_3_ in the (Al,Mn)O inclusions, so (Al,Y)O, (Al,Mn,Y)O, and (Mn,Y)O inclusions presented sequentially with an increase of Y content. The reactions are as follows:

1 $$2[{\rm{Y}}]+{{\rm{Al}}}_{2}{{\rm{O}}}_{3}({\rm{s}})\to {{\rm{Y}}}_{2}{{\rm{O}}}_{3}({\rm{s}})+2[{\rm{Al}}]\quad {{\rm{\Delta }}{\rm{G}}}^{{\rm{\theta }}}=-587482+270.28\,{\rm{T}}$$

2 $$2[{\rm{Y}}]+3{\rm{MnO}}({\rm{s}})\to {{\rm{Y}}}_{2}{{\rm{O}}}_{3}({\rm{s}})+3[{\rm{Mn}}]\quad {{\rm{\Delta }}{\rm{G}}}^{{\rm{\theta }}}=-909747+269.75\,{\rm{T}}$$

Figure 4b shows that MnS inclusions were generated at 0.004%–0.011% Y; the proportion of MnS inclusions would therefore increase after furnace cooling in this range of Y contents^25^.

### Electrochemical results

**Figure 5:**
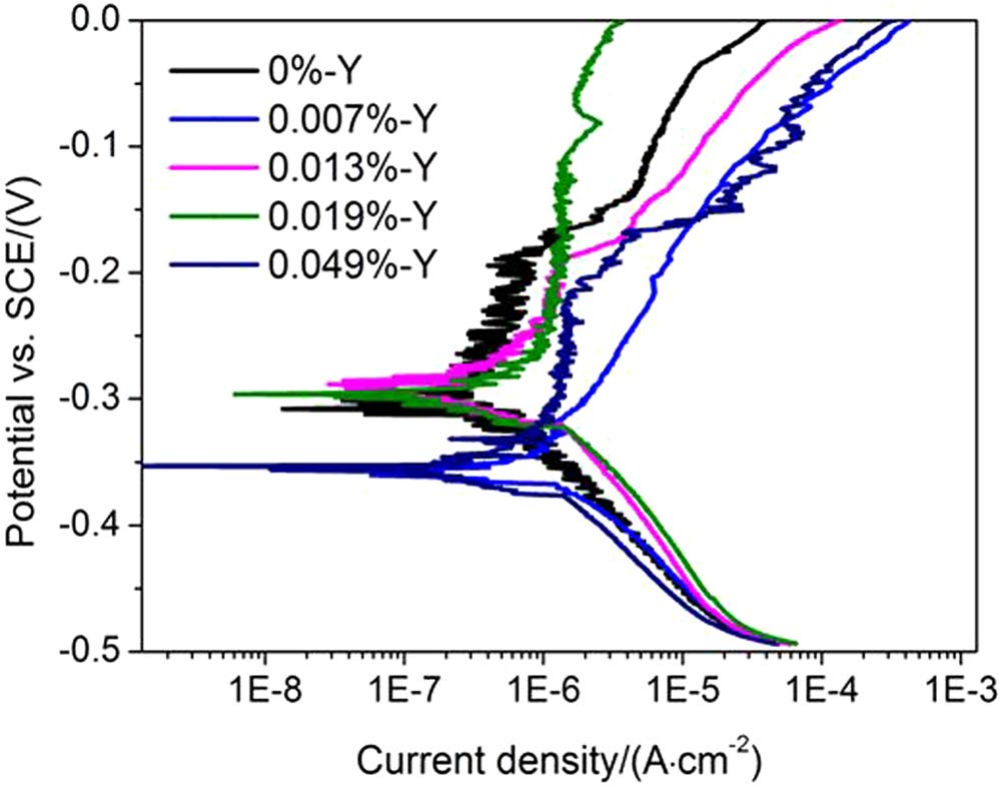
The potentiodynamic polarization curves of 304 stainless steel with different Y contents.

Potentiodynamic polarization tests were performed on the 304 stainless steels with different Y contents to determine their pitting corrosion resistance. The results are shown in Fig. 5; the corresponding corrosion potentials and pitting potentials are shown in Table 2. When the Y content was 0.007% or 0.049%, the corrosion potential (−0.35 V) was lower than for other Y contents, which indicated that severe pitting corrosion susceptibility occurred at these Y contents. The highest pitting potential (−0.11 V) was achieved by 0.019% Y, indicating that the 304 stainless steel with 0.019% Y possessed the best pitting corrosion resistance. Figure 5 shows that the pitting potential disappeared in 304 stainless steel containing 0.007% Y because the corrosion potential was greater than the pitting potential, so no passive region was attained: pitting corrosion occurred, indicating the weakest resistance to pitting corrosion by this steel composition. From these data, it was evident that the pitting potential first decreased, then increased, and then decreased again with increasing Y content.

**Table 2:** The corrosion potential and pitting potential in potentiodynamic polarization curves of 304 stainless steel with different Y contents.

Y contents	0%	0.007%	0.013%	0.019%	0.049%
corrosion potential/V	−0.31	−0.35	−0.29	−0.30	−0.35
pitting potential/V	−0.19	<−0.35	−0.18	−0.11	−0.22

### Characteristics of inclusions after immersion

**Figure 6:**
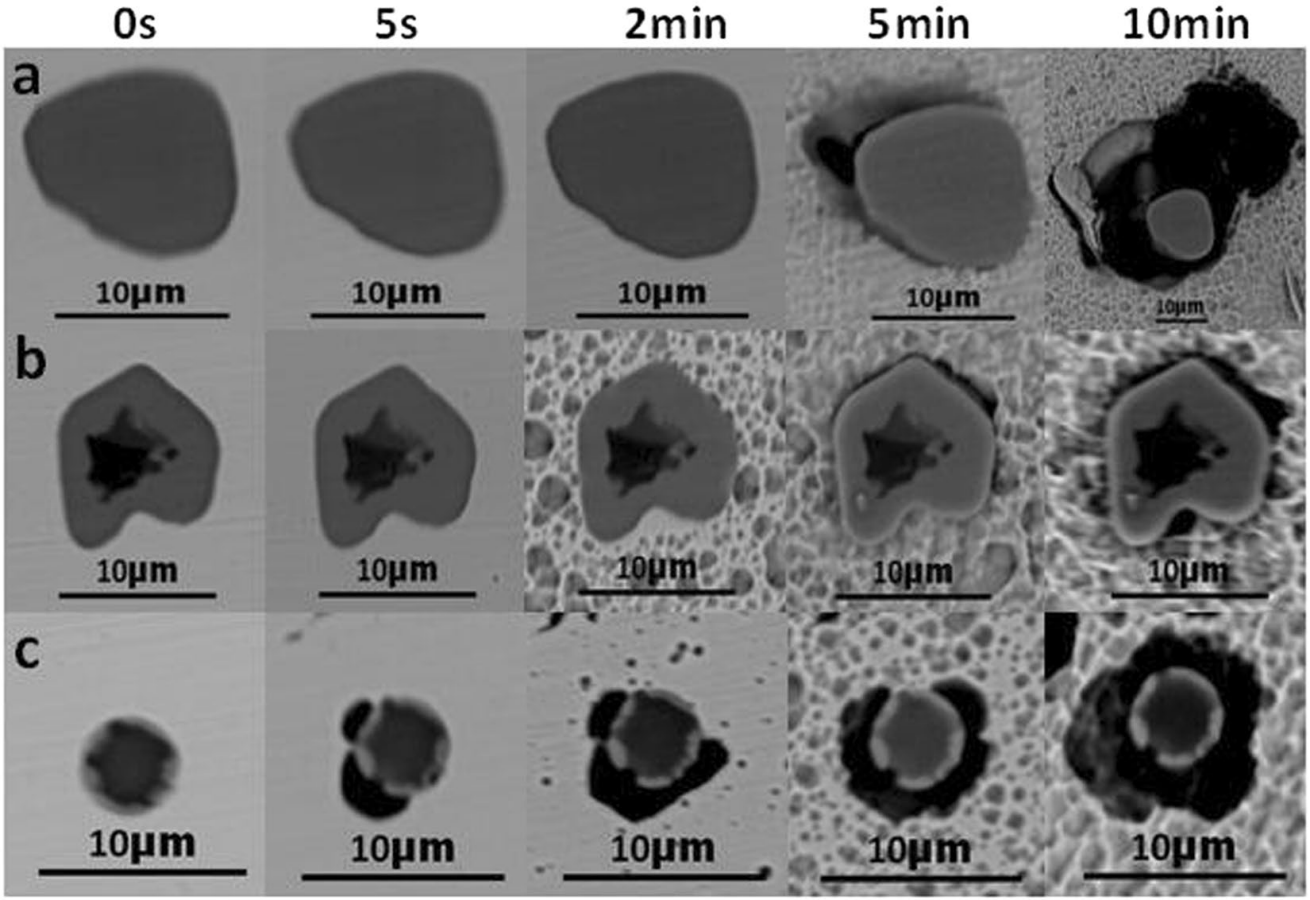
The morphology of (**a**,**b**,**c**) inclusions in 304 stainless steel containing 0% Y after immersion at 0 s, 5 s, 2 min, 5 min, 10 min. (**a**) (Al,Mn)O inclusions, (**b**) Al_2_O_3_ inclusions wrapped in (Al,Mn)O inclusions, (**c**) (Al,Mn,Si)O inclusions wrapped in (Al,Mn)O inclusions.

Immersion tests were conducted to observe the pitting of inclusions in 304 stainless steel with different Y contents and determine the effect of inclusion evolution on the pitting corrosion resistance. The morphologies of the inclusions in 304 stainless steel containing no Y after immersion for 0 s, 5 s, 2 min, 5 min, and 10 min are shown in Fig. 6, where **a** shows (Al,Mn)O inclusions, **b** shows Al_2_O_3_ inclusions wrapped in (Al,Mn)O inclusions, and **c** shows (Al,Mn,Si)O inclusions wrapped in (Al,Mn)O inclusions. Miro-crevices formed in the inclusion/matrix boundary at 5 min (Fig. 6a and b), while Fig. 6c shows the formation of a micro-crevice after 5 s.

**Figure 7:**
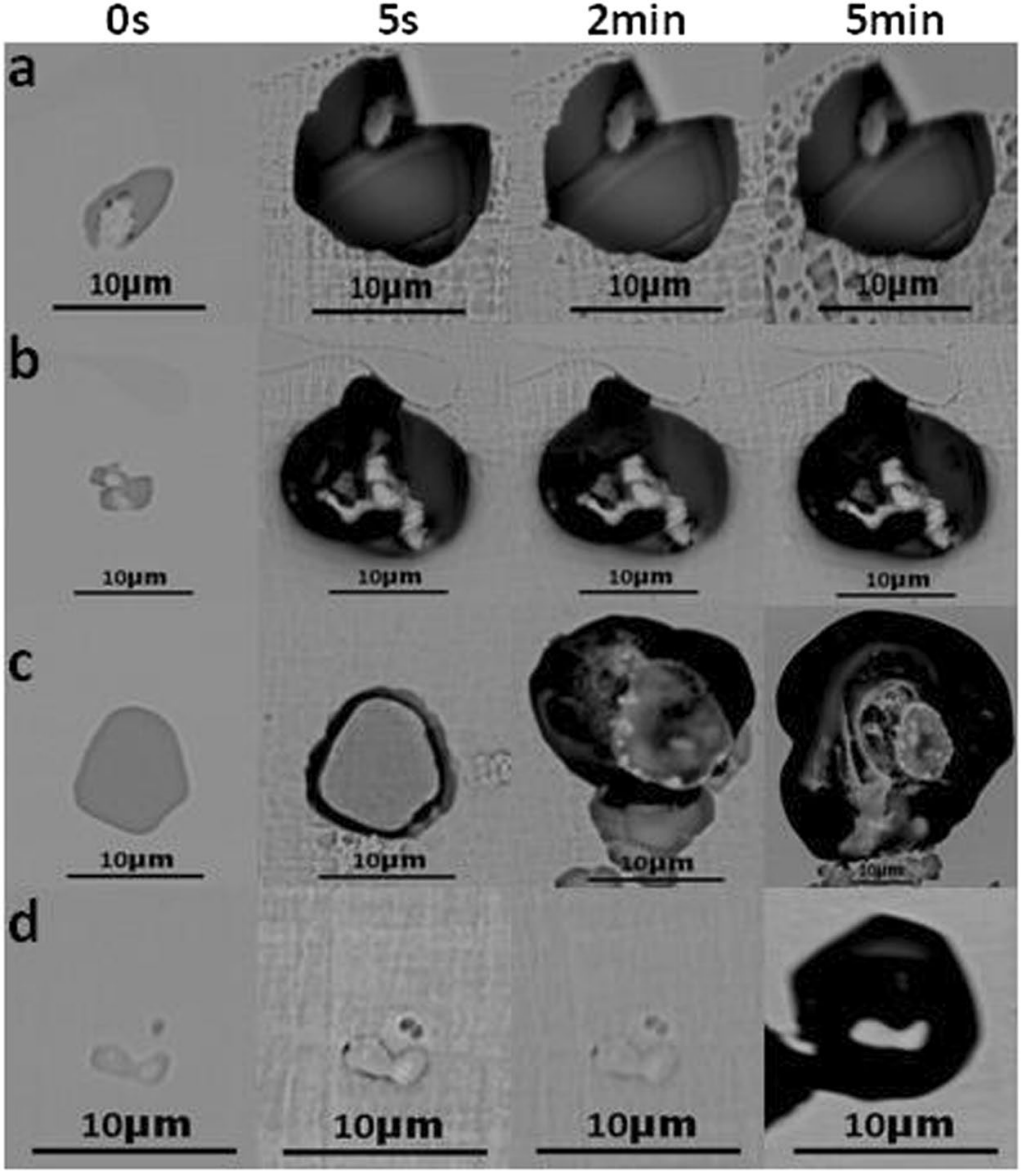
The morphology of (**a**,**b**,**c**,**d**) inclusions in 304 stainless steel containing 0.007% Y after immersion at 0 s, 5 s, 2 min, 5 min. (**a**) (Al,Y)_x_(SO)_y_ inclusions wrapped in MnS inclusions, (**b**) (Y,Mn)_x_(SO)_y_ inclusions wrapped in MnS inclusions, (**c**) MnS inclusions, (**d**) (Al,Y)O inclusions wrapped in (Al,Y)_x_(SO)_y_ inclusions.

The morphologies of inclusions in 304 stainless steel containing 0.007% Y after immersion for 0 s, 5 s, 2 min, and 5 min are shown in Fig. 7, where **a** shows (Al,Y)_*x*_(SO)_*y*_ inclusions wrapped in MnS inclusions, b shows (Y,Mn)_*x*_(SO)_y_ inclusions wrapped in MnS inclusions, c shows MnS inclusions, and d shows (Al,Y)O inclusions wrapped in (Al,Y)_*x*_(SO)_*y*_ inclusions. Figure 7a and b show serious corrosion, resulting in large pits after 5 s, while Fig. 7c shows the formation of a micro-crevice after 5 s and the development of corrosion along the inclusion/matrix boundary. The inclusion in Fig. 7d exhibited the best pitting corrosion resistance: serious corrosion only occurred after 5 min.

**Figure 8:**
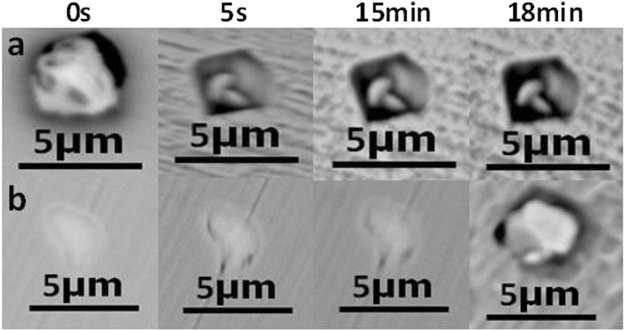
The morphology of (**a**,**b**) inclusions in 304 stainless steel containing 0.013% Y after immersion at 0 s, 5 s, 15 min, 18 min. a: Irregular-Y_2_O_3_ inclusions, b: Regular-Y_2_O_3_ inclusions.

**Figure 9:**
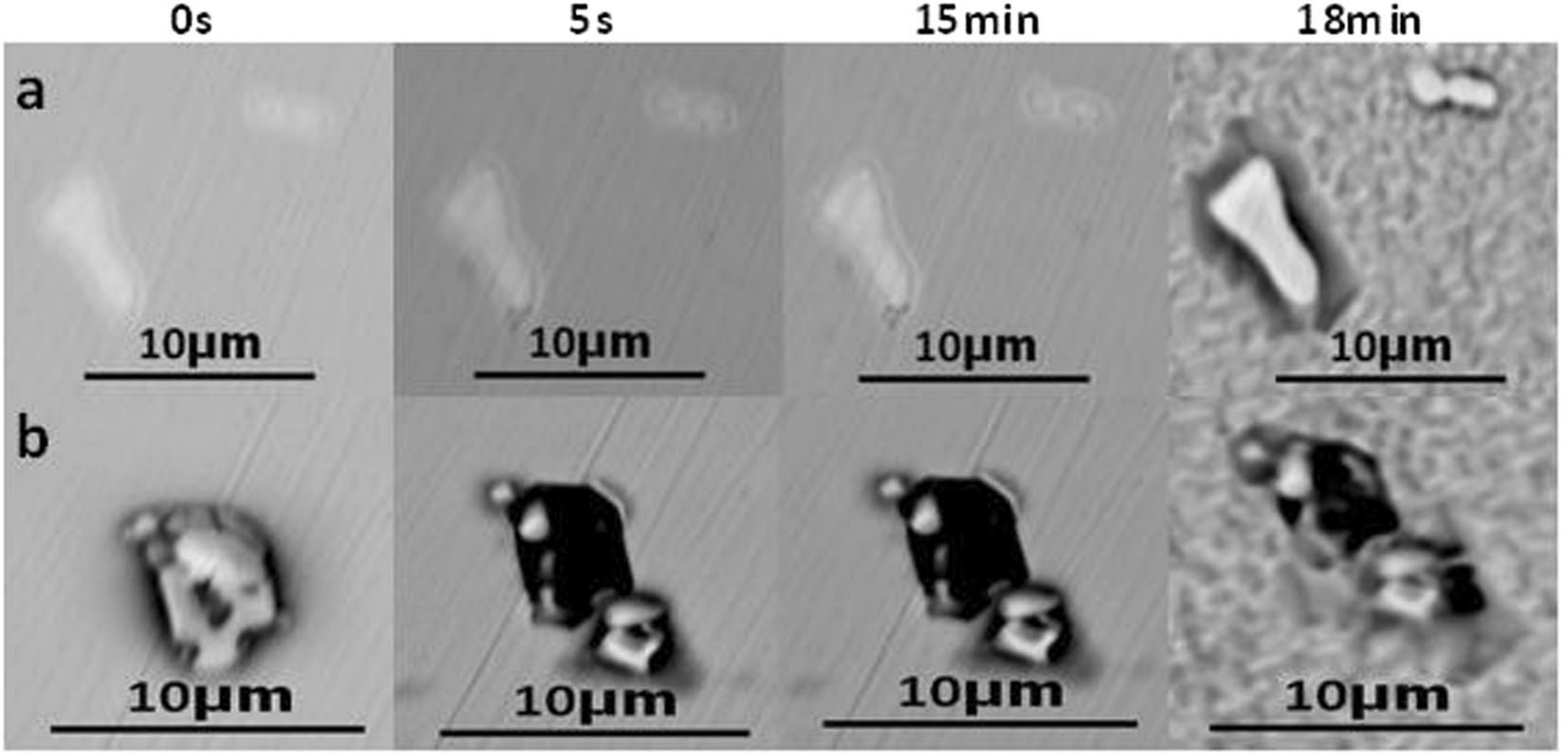
The morphology of (**a**,**b**) inclusions in 304 stainless steel containing 0.019% Y after immersion at 0 s, 5 s, 15 min, 18 min. (**a**) Regular-Y_2_O_3_ inclusions, (**b**) Irregular-Y_2_O_3_ inclusions.

The morphologies of inclusions in 304 stainless steel containing 0.013% Y after immersion for 0 s, 5 s, 15 min, and 18 min are similarly shown in Fig. 8, where a and b represent irregular and regular Y_2_O_3_ inclusions, respectively. Figure 8a shows pit formation after 5 s, the depth of which increased with time, while b showed no dissolution until 18 min. This trend is similar to that shown in Fig. 9, which represents 304 stainless steel containing 0.019% Y, where Fig. 9a and b show regular and irregular Y_2_O_3_ inclusions, respectively.

**Figure 10:**
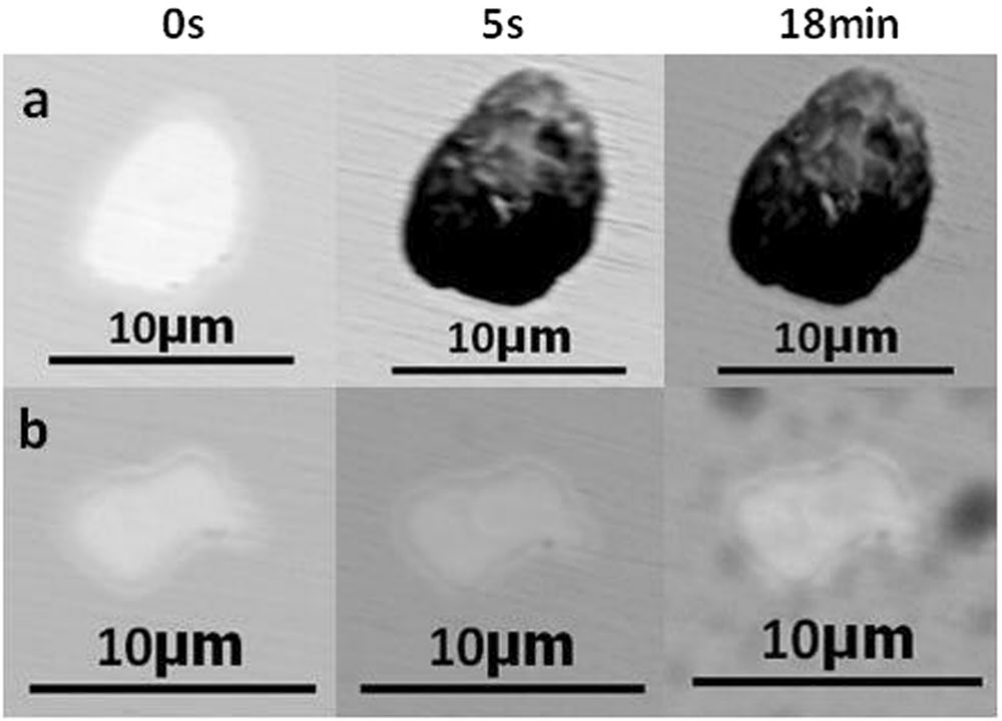
The morphology of a, b inclusions in 304 stainless steel containing 0.049% Y after immersion at 0 s, 5 s, 18 min. a: YN inclusions, b: Regular-Y_2_O_3_ inclusions.

The inclusion morphologies in 304 stainless steel containing 0.049% Y after immersion for 0 s, 5 s, and 18 min are shown in Fig. 10, where a represents YN inclusions and b shows regular Y_2_O_3_ inclusions. Figure 10a shows that large pits formed after 5 s, while b exhibited little or no corrosion after 18 min. This indicated that lower pitting corrosion resistance was achieved by 304 stainless steel containing 0.049% Y because of the generation of YN inclusions.

Pitting corrosion is randomly induced in stainless steel^26,27^. We therefore calculated the proportions of pits induced by inclusion after immersion for 5 s, 5 min, and 18 min in 304 stainless steel with different Y contents, as shown in Table 3. The results showed that different types of inclusions induced different trends of pitting initiation at these times. Pitting initiation induced by regular Y_2_O_3_ inclusions did not all occur after 18 min, which indicated that these inclusions had the best pitting corrosion resistance.

**Table 3:** The proportion of pits induced by inclusions at 5 s, 5 min, 18 min in 304 stainless steel with different Y contents.

Y contents/%	Inclusion number^a^	5 s	5 min	18 min
0	1	0%	94%	100%
	2	92%	100%	100%
0.007	8	82%	100%	100%
	4	88%	100%	100%
	7	100%	100%	100%
0.013	10	100%	100%	100%
	11	0%	0%	96%
0.019	10	100%	100%	100%
	11	0%	0%	92%
0.049	10	100%	100%	100%
	11	0%	0%	94%
	12	98%	100%	100%

^a^1: (Al,Mn)O inclusions, 2: (Al,Mn,Si)O inclusions wrapped in (Al,Mn)O inclusions, 4: (Al,Y)O inclusions wrapped in (Al,Y)_x_(SO)_y_ inclusions, 7: (Y,Mn)_x_(SO)_y_ inclusions wrapped in MnS inclusions, 8: MnS inclusions, 10: Irregular-Y_2_O_3_ inclusions, 11: Regular-Y_2_O_3_ inclusions, 12: YN inclusions.

## Discussion

The evolution of inclusions in 304 stainless steel with different Y contents directly affected their pitting resistance. The inclusion size first decreased and then increased with increasing Y content (Fig. 2). When the 304 stainless steel contained 0.013% Y, the average inclusion size in the matrix was lowest, but its pitting corrosion resistance was weaker than that of 304 stainless steel containing 0.019% Y (Fig. 5). Kim *et al*. argued that the addition of rare-earth elements changed the composition and shape of inclusions and decreased the inclusion size in duplex stainless steel^4^. Ha *et al*. illustrated that rare-earth elements reduced the size and surface density of (Mn,Cr,RE)-oxysulfide inclusions^20^ and that smaller inclusion sizes enhanced pitting corrosion resistance. From the point of this study, however, the type of inclusion (Figs 5–10) was more dominant than the inclusion size. With the increase of Y content, inclusions that were mainly composed of (Al,Mn)O and (Al,Mn,Si)O wrapped in (Al, Mn)O evolved into inclusions of MnS, (Al,Y)O wrapped in (Al,Y)_*x*_(SO)_*y*_, and (Y,Mn)_*x*_(SO)_*y*_ wrapped in MnS (0.007% Y), and then into Y_2_O_3_ inclusions. When the Y content increased further (0.013% to 0.019%), the proportion of regular Y_2_O_3_ inclusions increased further and YN inclusions were eventually generated (Figs 2–4). This evolution behaviour ensured that 304 stainless steel containing 0.007% Y gave the weakest resistance to pitting corrosion, because most inclusions (the composite inclusions) were etched and almost half were severely etched (Fig. 7). A large number of YN inclusions were severely etched after 5 s in 304 stainless steel containing 0.049% Y, but its pitting corrosion resistance was greater than the steel containing 0.007% Y because of the generation of regular Y_2_O_3_ inclusions (Fig. 10). The best pitting corrosion resistance was exhibited by 304 stainless steel containing 0.019% Y, which contained the largest proportion of regular Y_2_O_3_ inclusions (Figs 5, 9).

## Conclusions

In 304 stainless steel with Y addition, pitting corrosion resistance induced by inclusion was not predominantly related to the inclusion size, but also to the inclusion type. The composite inclusions and irregular Y_2_O_3_ inclusions showed the weakest resistance to pitting corrosion.

The pitting corrosion resistance first decreased, then increased, and finally decreased again with increasing Y content (within the range of 0%–0.049% Y) in 304 stainless steel. MnS inclusions and the composite inclusions were produced when Y contents were relatively low and YN inclusions formed at relatively high Y contents, both of which deteriorated the pitting corrosion resistance. Regular Y_2_O_3_ inclusions gave the best pitting corrosion resistance: the higher the number of inclusions, the better was the pitting corrosion resistance. The best pitting corrosion resistance was exhibited by 304 stainless steel containing 0.019% Y.
